# Nonlinear Absorption Response Correlated to Embedded Ag Nanoparticles in BGO Single Crystal: From Two-Photon to Three-Photon Absorption

**DOI:** 10.1038/s41598-018-20446-6

**Published:** 2018-01-31

**Authors:** Rang Li, Ningning Dong, Feng Ren, Hiro Amekura, Jun Wang, Feng Chen

**Affiliations:** 1 School of Physics, State Key Laboratory of Crystal Materials, Shandong University, Jinan, 250100 China; 2 Key Laboratory of Materials for High-Power Laser, Shanghai Institute of Optics and Fine Mechanics, Chinese Academy of Science, Shanghai, 201800 China; 3 Department of Physics, Center for Ion beam Application and Center for Electron Microscopy, Wuhan University, Wuhan, 430072 China; 4 National Institute for Materials Science, 3-13 Sakura, Tsukuba, Ibaraki, 305-0003 Japan

## Abstract

We report on the embedded silver (Ag) nanoparticles fabricated by Ag^+^ ion implantation into the Bi_4_Ge_3_O_12_ (BGO) crystal. Localized surface plasmon resonance (LSPR) phenomenon has been observed by linear optical absorption spectrum, which is accordance with the expectation based on Mie theory calculation. Further proofs are given by SRIM, TEM and SAED analysis, which explain the slight difference between experiment and calculation. Based on the z-scan system, it is found that the nonlinear optical response is converted from two-photon absorption to three-photon absorption under the 515 nm femtosecond pulse excitation within the LSPR band. The nonlinear absorption coefficient is measured to be ~3.1 × 10^−9^ cm/W (two-photon absorption coefficient) and ~8.9 × 10^−14^cm^3^/W^2^ (three-photon absorption coefficient) for pure BGO crystal and the sample embedded with Ag nanoparticles (Ag:BGO), respectively. Finally, we have proposed a model to explain the asymmetric nonlinear transmittance, which is in good agreement with the experimental results.

## Introduction

In the recent decades, techniques based on multi-photon absorption have been developed to achieve a number of photonic applications such as optical power limiter^1,2^, up-conversion lasing^3,4^, data storage^5,6^, and biological imaging^7,8^, due to the correlated effects acting on diverse systems^9–12^. Since the pioneering work by Maria Goeppert-Mayer in 1931, in which the multi-photon absorption mechanism was proposed for the first time, materials with high order nonlinear optical responses have been one of the focuses for researchers^13–16^. Compared with the single-photon absorption and two-photon absorption, three-photon absorption owns more compact spatial selectivity because the criterion of three-photon absorption is much stricter: Only in the very tiny region around the laser focus, the optical intensity could exceed threshold. In spite of the higher order nonlinear optical response has prior properties, three-photon absorption phenomenon is much easier to be observed in the scientific experiment. In practice, three-photon absorption often occurs in the organic materials or in the near infrared wavelength region^17–20^, which limits the development of the nonlinear optical application. In this way, nonlinear optical response from two-photon absorption to three-photon absorption in dielectric crystals may be of considerable significance towards diverse applications.

Metallic nanoparticles have been one of the most popular topics of the nonlinear nanocomposite materials ascribed to its surface plasmon resonance and quantum size effect^21,22^. The excellent nonlinear optical responses of the noble metallic nanoparticles have been reported in many previous literatures^23–25^, and most of which are mainly using chemical methods to synthesize nanoparticles^26–28^. Nevertheless, ion implantation shows a unique advantage that it enables nanoparticles to be embedded in the target matrix which has a particular dielectric environment Furthermore, types and sizes of nanoparticles have the ability to be modulated by the species and fluences of implanted ions flexibly, and almost any desired materials could be selected as substrate during the implantation process in theory^29,30^. However, to the best of our knowledge, there is no such a work which realized the fabrication of nanoparticles by ion implantation in BGO crystals and few researches were reported even the field expanded to the crystals. As we all know, numerous studies have shown the significant enhancement of optical nonlinearity on account of the collective oscillation of electron gas that couples with electromagnetic fields, and most of these work are devoted to the saturated absorption^31–33^. In consequence, the nonlinear optical properties of the nanocomposite combined by the noble metal nanoparticles and BGO crystals are well deserved to be investigated.

As a novel generation of scintillation crystals^34^, BGO shows the superior characteristics including high density, well chemical resistance and high mechanical strength, which make it utilized in high energy physics experiments like the large electron-positron collider in CERN. Both experimental and theoretical results have demonstrated the two-photon absorption property of pure BGO crystals under the high power laser at the wavelength of visible waveband^35,36^. Compared with conventional nonlinear optical crystals, BGO exhibits three- and five-order nonlinear absorption response in the visible and infrared optical band, respectively. It performed as well as the other excellent nonlinear optical materials in the nonlinear optical field due to its large nonlinear coefficient. Besides, as the typical photorefractive crystal, BGO crystal is of certain interest in view of its diverse application in optoelectronics and laser physics, such as four-wave frequency mixing and optical switching. In this work, we report on the synthesis of Ag nanoparticles embedded in BGO crystals by using Ag^+^ ion implantation. Experiment and calculation of the linear absorption spectra both prove the formation of Ag nanoparticles which is further confirmed and explained by SRIM, TEM and SAED analysis. We observe an efficient surface modification through the asymmetric nonlinear transmittance by z-scan measurement with femtosecond pulses at 515 nm within the SPR band. Discussion is made in detail which comes to the conclusion that the two-photon absorption is converted to three-photon absorption by the embedded Ag nanoparticles in BGO crystal.

## Results and Discussion

**Figure 1:**
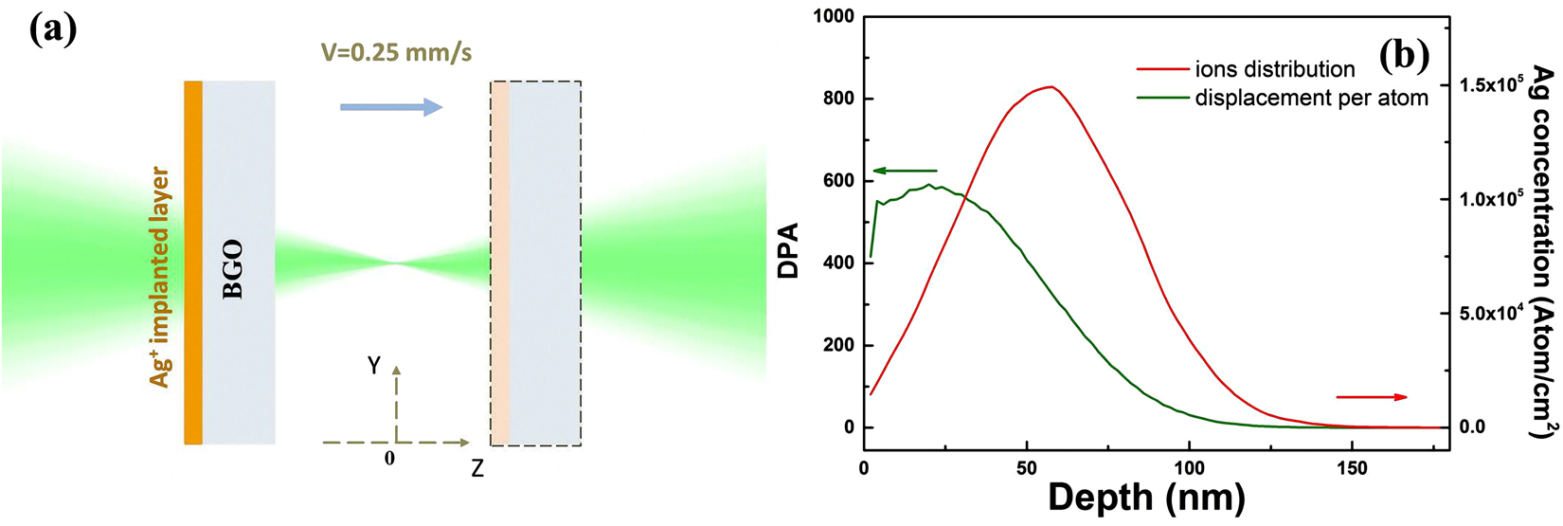
Z-scan schematic diagram and SRIM simulation. (**a**) Schematic of z-scan measurement of Ag^+^ implanted BGO crystal. (**b**) The Ag^+^ ions distribution (in red); the displacement per atom distribution(in green).

Figure 1(b) demonstrates the simulation results of the ion distribution and displacement per atom (DPA) distribution by SRIM with a hundred thousand numbers of Ag^+^ ions. The distribution of Ag^+^ ions presents a Gauss-like distribution property with the range from the surface of the sample to the 130 nm depth below. As we can see, the center of the Ag ions distribution located at ~60 nm with the concentration of more than 1.5 × 10^5^ Ag^+^/cm^2^, which would be 1.5 × 10^17^ atoms/cm^2^ when the fluence is 1 × 10^17^ ions/cm^2^ in our case. In this way, Ag nanoparticles are formed after implantation for the reason that the concentration of Ag atoms is in excess of the solubility limit of the substrate and these implanted atoms aggregate to form nanoclusters spontaneously. Besides, the DPA distribution in Fig. 1(b) shows that the lattice damage is much more serious at the surface of the sample. The maximum value of DPA is as high as 400, which could be large enough to make the implanted layer amorphous.

**Figure 2:**
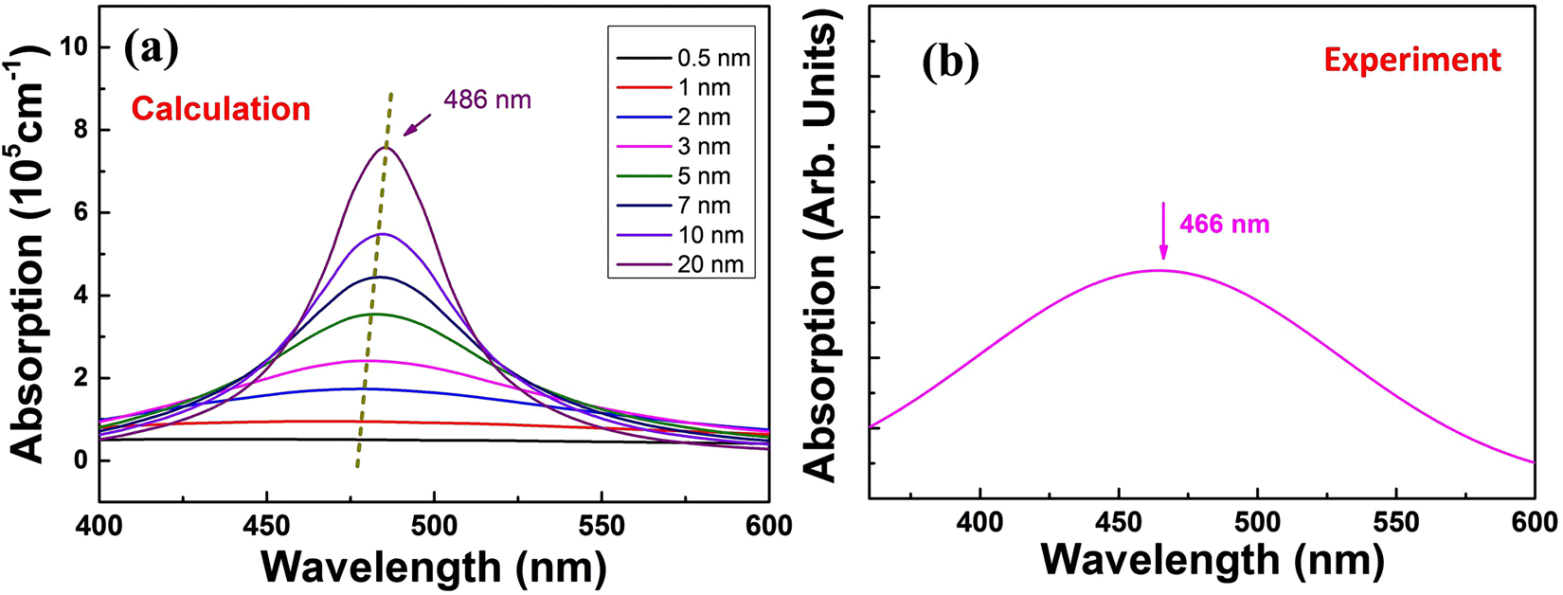
Linear absorption properties of the implanted samples. (**a**) Calculated absorption of Ag nanoparticles by Mie theory and (**b**) experimental absorption spectrum of the Ag:BGO sample.

Figure 2(a) and (b) depict the linear optical absorption characterization of Ag nanoparticles theoretically and experimentally. Using the Mie theory with the formula^37^,

1 $$\gamma =\frac{18\pi p{\varepsilon }_{d}^{3/2}}{{\lambda }_{0}}\frac{{\varepsilon }_{m}^{^{\prime\prime} }}{|{\varepsilon }_{m}+2{\varepsilon }_{d}{|}^{2}}$$

where ${\varepsilon }_{m}$ and ${\varepsilon }_{d}$ are the complex dielectric constants of metal and insulator respectively. ${\varepsilon }_{m}^{^{\prime\prime} }$ and ${\lambda }_{0}$ denote the imaginary part of ${\varepsilon }_{m}$ and the wavelength of light in vacuum. *p* represents the volume fraction of metal. It should be noted that the traditional refractive index of BGO crystal is not suitable in this case to calculate ${\varepsilon }_{d}$ according to the results by SRIM. The embedded Ag nanoparticles are distributed at the implanted layer which may become amorphous with a changed refractive index. It is recognized that the refractive index distribution of the implanted sample has always been tough to be measured and no previous work reported the refractive index distribution of Ag implanted BGO crystals. To solve this problem, we assume the refractive index of the implanted layer is changed with a similar trend (~10% decrease) as the well-learned implanted LiNbO_3_ crystals^38^. The results are displayed in Fig. 2(a). It is obvious that the absorption peak is located at 486 nm and it has a slight blue shift as the size of nanoparticles decreases from 20 nm to 0.5 nm. The size dependence is caused by the classical mean-free path confinement effect, which can be included in the calculation: ${\omega }_{tau}={\omega }_{tau,0}+\frac{2{v}_{F}}{d}$, where ${\omega }_{tau,0}$ denotes the bulk relaxation energy, ${v}_{F}$ denotes the Fermi velocity and $d$ is the diameter of the nanoparticles^37^. Figure 2(b) gives us the experimental absorption spectrum, whose peak is at around 466 nm. As far as we consider, there could be two reasons for the little difference between calculation and experiment results. Firstly, the nanoparticles in our samples are so small that its absorption peak has a blue shift due to the “size effect” as the results of calculation show^37^. Secondly, the implanted layer is not completely amorphous that the refractive index we use is not accurate.

**Figure 3:**
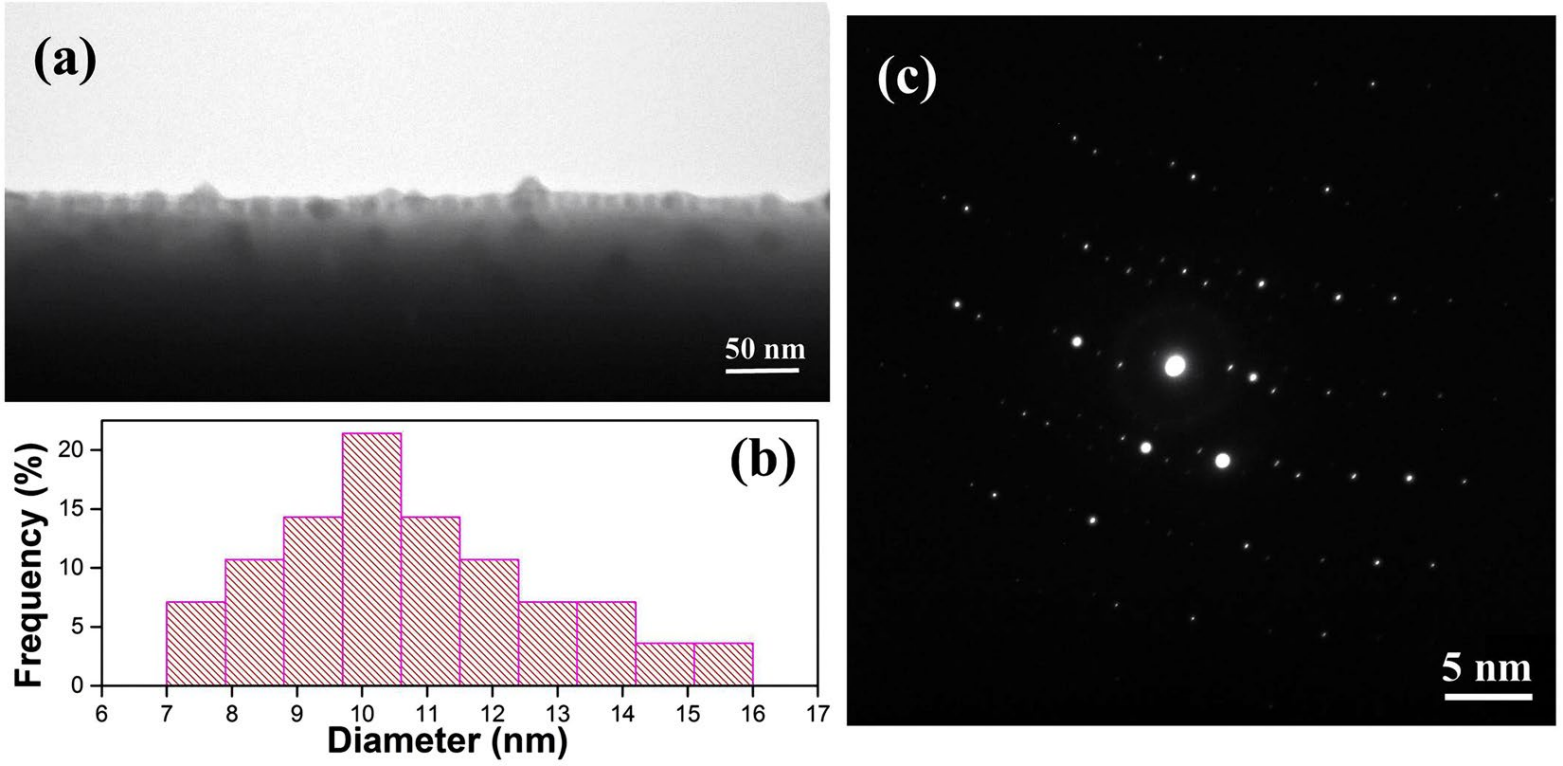
TEM and SAED analysis. (**a**) Superficial overview bright-field TEM micrograph of the Ag:BGO sample. (**b**) Statistical analysis of the TEM image. (**c**) SAED analysis of the Ag:BGO sample.

To verify our speculation, further work is accomplished by transmission electron microscopy and selected area electron diffraction analysis. Figure 3(a) shows a superficial overview cross-sectional TEM micrograph of the Ag:BGO sample, which confirms the formation of the Ag nanoparticles intuitively. The statistical analysis of the TEM image shown in Fig. 3(b) indicates the mean diameter of nanoparticles is ~10.7 nm and the standard deviation is ~2.1 nm. Figure 3(c) exhibits the polycrystalline state of the implanted layer, which demonstrates the refractive index we used could have a little deviation with the real one. Considering the size effect is weak and most of the nanoparticles are much larger than 7 nm, the amorphous degree should be the main cause of the difference between calculation and experiment. It also reveals a potential application for detecting the amorphous degree of film by simple absorption measurement of nanoparticles. Further work will be expected but it’s not the key point of this article.

The excitation pulse energy dependent nonlinear optical response characterization is investigated by z-scan system with an open aperture. When it comes to multi-photon absorption effect, traditional z-scan theory should be corrected accordingly. The propagation equation in these samples can be written as: $$dI/d{z}^{\text{'}}=-{\alpha }_{0}I$$$$-\sum _{m=2}{\alpha }_{m}{I}^{m}-{\sigma }_{a}{N}_{e-h}I$$, where *I* is the excitation intensity, *z*′ is the propagation distance in the samples, $${\sigma }_{a}$$ is the cross term of absorption and refraction by the photo-excited charge carriers, $${N}_{e-h}$$ is the number density of the photo-excited charge carriers, *α*_0_ is the linear absorption coefficient and *α*_*m*_ is the nonlinear absorption coefficient. This equation can be solved as follows^39^,

2 $${T}_{Norm}(z)=\frac{1}{{\pi }^{1/2}{q}_{0}}\int \mathrm{ln}\,[1+{q}_{0}\exp (-{x}^{2})]dx$$

3 $${T^{\prime} }_{Norm}(z)=\frac{1}{{\pi }^{1/2}{p}_{0}}\int \mathrm{ln}\{{[1+{p}_{0}^{2}\exp (-2{x}^{2})]}^{1/2}+{p}_{0}\exp (-{x}^{2})\}dx$$

where${q}_{0}={\alpha }_{2}({I}_{0}{L}_{eff})/(1+{z}^{2}/{z}_{0}^{2})$, ${p}_{0}={(2{\alpha }_{2}{L^{\prime} }_{eff})}^{1/2}({I}_{0})/(1+{z}^{2}/{z}_{0}^{2})$, ${L}_{eff}=[1-{e}^{-{\alpha }_{0}L}]/{\alpha }_{0}$, ${L^{\prime} }_{eff}=[1-{e}^{-2{\alpha }_{0}L}]/2{\alpha }_{0}$. ${L}_{eff}$ and ${L^{\prime} }_{eff}$ is the effective thickness of the Ag NPs layer at the condition of two-photon and three-photon absorption respectively. *L* is the Ag NPs layer thickness, *I*_0_ is the light intensity at the focus and *z*_0_ is the beam’s diffraction length. After a further approximation and simplification, formulas (2) and (3) can be written as^40^:

4 $$\mathrm{ln}(1-{T}_{Norm}(z))=\,\mathrm{ln}(I(z))+C$$

5 $$\mathrm{ln}(1-{T^{\prime} }_{Norm}(z))=2\,\mathrm{ln}(I(z))+C^{\prime} $$

where $C$ and $C^{\prime}$ are the constants. Formulas (2) and (4) are appropriate for the two-photon absorption while the expressions (3) and (5) are suitable for the three-photon absorption condition, from which we can easily distinguish the order of the nonlinear optical response by ln(1-*T*(*z*)) vs ln(*I*(*z*)).

**Figure 4:**
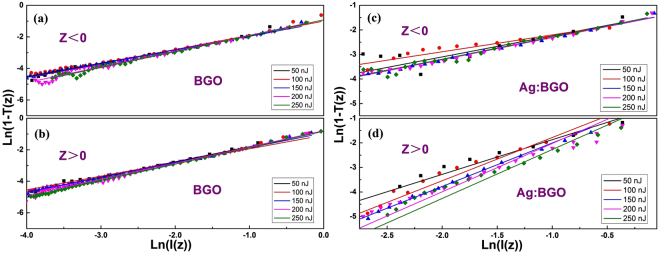
The judgment of the orders of the nonlinearity. Plots of ln(1-*T*(*z*)) vs ln(*I*(*z*)) of BGO when (**a**) *z* < 0 and (**b**) *z* > 0; Plots of ln(1-*T*(*z*)) vs ln(*I*(*z*)) of Ag implanted BGO when (**c**) *z* < 0 and (**d**) *z* > 0.

Figure 4 exhibits the plots of ln(1-*T*(*z*)) vs ln(*I*(*z*)) of BGO crystal and Ag:BGO sample at various excitation energies. The solid lines are linear fittings to the experimental data, whose slopes are displayed in Table 1. It is intriguing that the nonlinear absorption responses on both sides of the laser focus (*z* = 0) seem completely different. As we can see, within the margin of error, the slopes are all closed to 1.0 when *z* < 0, which indicates the two-photon absorption. Differently, when *z* > 0, the slopes equal to 2.0 at the high energy for Ag:BGO sample which indicates the three-photon absorption, but the value is 1.0 still for pure BGO crystal. In addition, the slope value (such as 1.4) between 1.0 and 2.0 at low energy implies that both the two-photon absorption and three-photon absorption are existed. The reason we believe is that it is caused by the delicate double-layers structure of the sample. As it can be seen in Fig. 1(a), the sample was moving from the forward (*z* < 0) to the backward of the laser focus (*z* > 0) during the experiment process and the intensity of the laser is much higher at the region near the laser focus. When *z* < 0, the substrate is much closer to the focus of laser than the Ag^+^ implanted layer, which means the nonlinear absorption response of the substrate plays a more important role due to the high intensity of laser. On the contrary, when *z* > 0, the position where the Ag^+^ implanted layer is, is nearer to the laser focus than where the substrate be. That is to say, the nonlinear absorption response of the implanted layer is dominant when the sample passes through the laser focus. Based on this model, we can make a conclusion that the implanted layer possesses a completely different nonlinear absorption response compared with the substrate. The two-photon absorption converts to three-photon absorption by the embedded Ag nanoparticles using the ion implantation technology. It should be noted that the observed three-photon absorption response is actually an effective three-photon absorption, which is arising from the two-photon absorption followed by excited state absorption^41,42^. Besides, the saturable absorption mechanism of Ag nanoparticles and the two-photon absorption of amorphous implanted BGO layer could also contribute to the obtained z scan results. The instantaneous and accumulative nonlinear effects take place together making the nanocomposite layer behaves the same nonlinear absorption effect as the pure three-photon absorption does.

**Table 1:** The value of ln(1-T(*z*))/ ln(*I*(*z*)) for BGO and Ag:BGO under different excitation energy.

Excitation Energy	BGO		Ag:BGO	
	*z* < 0	*z* > 0	*z* < 0	*z* > 0
50 nJ	0.9	1.0	0.9	1.4
100 nJ	0.9	0.9	0.7	1.8
150 nJ	0.9	0.9	0.9	1.8
200 nJ	1.0	1.0	0.9	2.0
250 nJ	1.0	1.0	0.9	2.0

**Figure 5:**
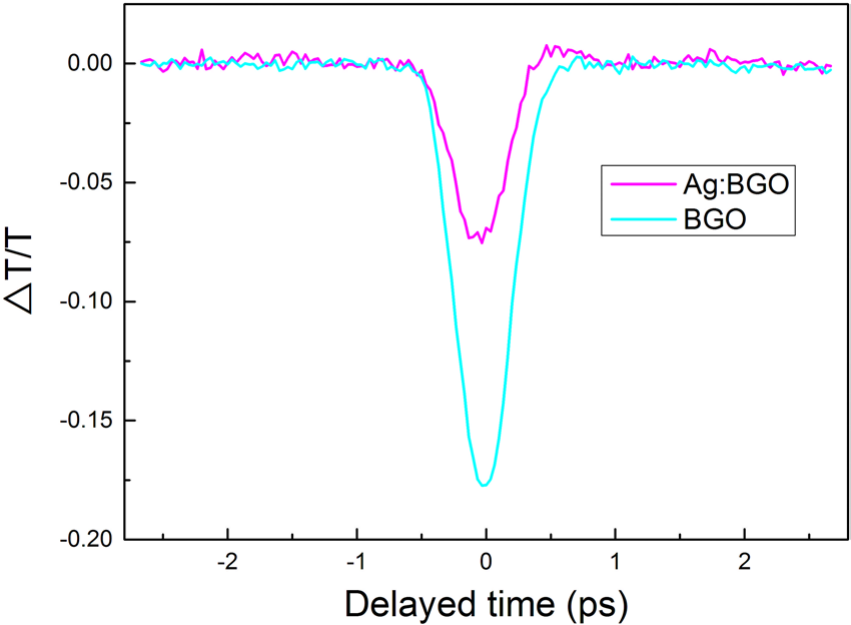
Time evolution of the normalized transmission change for BGO and Ag:BGO with an excitation of 515 nm and 340 fs pulses. The pink line and blue line represent the pump-probe results for Ag:BGO and BGO, respectively.

To verify the process is indeed an electronic excitation rather than other effects such as nonlinear scattering, laser induced damage or thermally induced shifting of the absorption, we did pump-probe studies for pure BGO and Ag implanted BGO. Typical results are shown in Fig. 5. We can see that the pure BGO crystal displays two-photon absorption after the zero-time-delay and the curves recover to the original value after 0.7 picosecond. For Ag:BGO, the pump-probe result indicates the two-photon absorption after zero-time-delay because of the combined contribution of Ag nanoparticles and implanted BGO layer. On one hand, the excited state absorption caused by free carriers (generated by two-photon absorption) happens in the nanoparticle system^41,42,44^; on the other hand, the two-photon absorption of the implanted BGO layer becomes much weaker due to the amorphous state. Besides, there is an un-obvious peak at the end of the valley, which indicates the SA mechanism is also exist but it is relatively weaker compared with the two-photon absorption mechanism and excited state absorption mechanism. In conclusion, the pump-probe results are in accordance with our opinion that the observed effective three-photon absorption is actually the multi-mechanisms induced nonlinear optical response. The two-photon absorption of implanted BGO layer, the saturable absorption and two-photon absorption of Ag nanoparticles and especially the excited state absorption of free carriers, all these factors make it a three-photon absorption like z scan result. Such a complicated system can also be seen in the ref.^43^. In this way, the nonlinearity can be considered as an effective three-photon absorption caused by electronic excitation, which can be well-applied to the traditional three-photon absorption photonic devices and broaden the application range of the two-photon absorption materials meanwhile.

**Figure 6:**
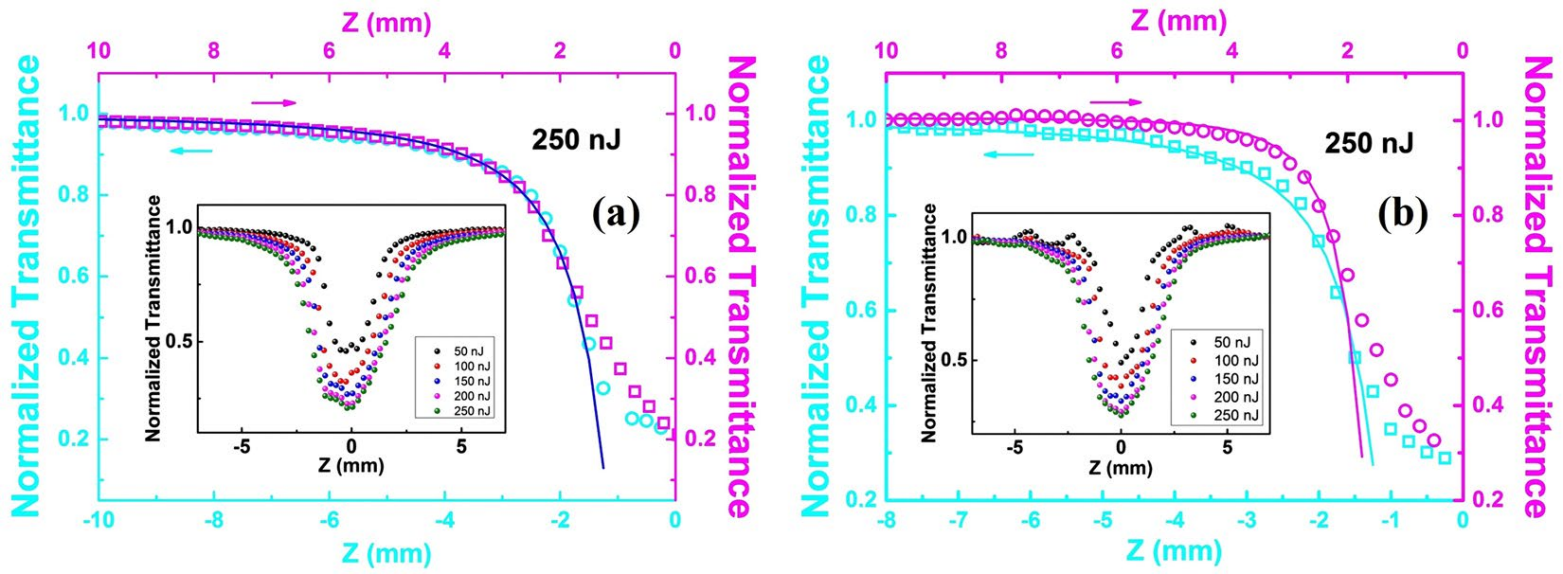
The nonlinear optical response property of BGO and Ag:BGO. Fitting lines to the normalized transmittance as a function of *z* at 250 nJ for (**a**) BGO crystal and (**b**) Ag:BGO sample respectively. The inserts depict the excitation energy dependence.

Figure 6 (a) and (b) are the fitting lines to the transmittance as a function of *z* at 250 nJ for BGO crystal and Ag:BGO sample respectively and the excitation energy dependence is depicted in the insert. In Fig. 6(a), both the conditions (*z* < 0 and *z* > 0) are well fitted by the formula (2), which indicates the two-photon absorption of BGO crystal. The two-photon absorption coefficient is obtained to be 3.1 × 10^−9^ cm/W with an error of ±15% and it is basically consistent with the results in the refs.^35,36^. The normalized transmittance varies smoothly with the laser intensity increasing at different excitation energies and the data points are symmetrical about the laser focus. Figure 6(b) shows the lines fitted by expression (2) and (3) for Ag:BGO sample at *z* < 0 (blue line) and *z* > 0 (pink line) respectively and it is obvious that the normalized transmittance decreases more steeply when *z* > 0 which presents the admirable optical limiting properties of three-photon absorption compared with the two-photon absorption. The effective three-photon absorption coefficient is 8.9 × 10^−14^ cm^3^/W^2^, which is ~six orders magnitude in comparison to that of pure BGO crystal at 1064nm^35^. When *z* < 0, the calculated two-photon absorption coefficient makes almost no difference to the value of the BGO crystal. Fittings under the conditions of low excitation energy is not accomplished because of the combined effect of two-photon absorption and three-photon absorption, which can be seen in the insert image. Especially the energy is modulated to be 50 nJ, the curve seems undulant due to the multiple absorption responses, which can also be proved by Table 1. It should be mentioned that these fitting lines fail to fit the data near the laser focus within a small range from *z* = −1.7 to *z* = 1.7 mm. Referring to the reported optical damage thresholds of BGO crystals, we believe the BGO crystal is partly damaged by the high intensity near the laser focus. The peak intensity at the position *z* = 1.7 mm is ~3 × 10^23^ W/cm^2^, which is exactly in accordance with the calculated peak intensity according to the data in ref.^36^ assuming its pulse width is 10 ps. Future work would be focused on the perfection of the explanation model in detail to take the multiple complicated interferences into consideration and we are looking forward to realizing the potential application of the modulation of the nonlinear absorption response by embedded nanoparticles in crystals.

## Conclusions

In conclusion, Ag nanoparticles embedded in BGO crystals has been realized by ion implantation. With an average diameter of ~10.7 nm, the morphology properties of the nanoparticles are characterized by TEM observation. Linear optical absorption has been obtained experimentally and theoretically, and the slight difference is mainly caused by the partial amorphization of the implanted layer, which is analyzed by SRIM and SAED. Using z-scan system, the nonlinear optical response is investigated by comparing the results of implanted sample and non-implanted sample. It is believed that the nonlinear optical response of the implanted layer is demonstrated to be effective three-photon absorption, which is further discussed in details by a preliminary explanation model. The nonlinear absorption coefficients are measured to be ~3.1 × 10^−9^ cm/W (two-photon absorption coefficient) and ~8.9 × 10^−14^cm^3^/W^2^ (effective three-photon absorption coefficient) for pure BGO crystal and the sample embedded with Ag nanoparticles, respectively. The latter value is ~6 orders magnitude higher in comparison to the three-photon absorption coefficient of pure BGO crystal at 1064 nm, which exhibits the wide potential application in photonic devices like data storage and optical limiting, etc.

## Methods

### Synthesis of Ag nanoparticles

The Ag nanoparticles are fabricated in an optically polished BGO crystal, which is cut with dimension of 10 mm × 10 mm × 2 mm, by using the analytical type ion implanter LC22-1C0-01 at Wuhan University, China. The 200 keV Ag^+^ ion beam, with a fluence of 1 × 10^17^ ions/cm^2^, was titled by 7° off the vertical plane of the sample surface to avoid the channeling effect.

### SRIM simulation

The Ag^+^ ion distribution and the displacement per atom (DPA) were calculated by the software Stopping and Range of Ions in Matter (SRIM-2011) code. The implanted ions are set as Ag with the energy of 200 keV and the target is set as Bi_4_Ge_3_O_12_ with the density of 7.13 g/cm^3^. The total number of the ions simulated was 100,000 for the purpose of accuracy.

### Characterization of the SPR absorption

The UV-VIS-NIR spectrophotometer (Shimadzu, UV-1800) is employed to obtain the absorption spectrum which is compared with the results based on Mie theory. The slit is 1 nm and the scanning speed is set as middle speed in the experiment. The wavelength of the probe light changes from 300 nm to 600 nm.

### TEM and SAED analysis

For the purpose of observing the spatial distribution, shape and size of the Ag nanoparticles, transmission electron microscopy (TEM) analysis was carried out using JEM 2010 (HT) microscope operated at an accelerating voltage of 200 kV. The statistics of the size distribution of Ag nanoparticles were analyzed by Nano Measurer. The selected area electron diffraction (SAED) measurement was also obtained to investigate the amorphization degree of the implanted film.

### Nonlinear optical response measurement

As it can be seen in Fig. 1(a), the nonlinear optical response properties are studied by z-scan system with an open aperture. With our sample moving through the focus of the lens along the z axis (laser propagation direction) with a speed of 0.25 mm/s, the transmittance can be measured as a function of *z*. The wavelength of the excitation laser is 515 nm with a repetition rate of 100 Hz and pulse width of 340 fs. The focal length of the lens is ~15 cm and the beam waist radius is estimated to be ~15 μm at the focus. The average energy of the probe laser is changing from 50 nJ to 250 nJ with an increment of 50 nJ.

### Time-resolved pump-probe experiment

The transient optical experiments of BGO and Ag:BGO were carried out using the pump-probe method with a femtosecond laser system (515 nm, 340 fs, 100 Hz). The laser pulses were split into two beams as pump and probe. The pump energy was measured to be 2.9 μJ and the probe energy was 35 nJ. After that, the pump and probe beam were focused on the sample and the beam spot radii at the focus was estimated to be 125 μm. By changing delay of the probe pulses by the motorized stage, we obtained the curves as a function of probe delay with the change in transmission. The polarization of the pump beam was kept perpendicular to the probe beam throughout all the experiments.
